# Unusual application of twinkling artifact

**DOI:** 10.1007/s40477-023-00861-w

**Published:** 2024-06-19

**Authors:** Francesco M. Drudi, Roberto Maroncelli, Flavia Angelini, Maurizio Renda, Gianluca Maglia, Michele Bertolotto, Vito Cantisani

**Affiliations:** 1 Department of Radiology, University La Sapienza, Viale Regina Elena 324, 00161 Rome, Italy; 2 Department of Radiology, University of Trieste, Trieste, Italy

**Keywords:** CDUS, Twinkling artifact, Foreign bodies, US grayscale

## Abstract

**Introduction:**

The aim of this paper is to show how to improve diagnostic accuracy using CDUS and twinkling artifact in patients experiencing discomfort due to the presence of small FBs in the soft tissues not clearly visible at US grayscale examination.

**Materials and methods:**

We enrolled 7 adult patients presenting with small (2–4 mm) superficial FBs located in the subcutaneous and muscle tissues, barely or not detectable on US grayscale. All patients underwent US grayscale and CDUS examinations.

**Results:**

We identified superficial FB with twinkling artifact in all 7 patients. All of these were confirmed to represent foreign bodies after surgical excision.

**Conclusion:**

TA is useful in the evaluation of subcutaneous and muscular FBs and provides information on their location, depth and shape, which is useful if surgical excision is required.

## Introduction

Twinkling artifact (TA) is a color Doppler ultrasound (CDUS) artifact consisting of a fluctuating artefactual color mix of rapidly changing red and blue vertical bands behind a highly reflective, granular structure composed of several reflecting elements. On the contrary, TA does not occur behind smooth reflective structures [1].

TA occurs due to erroneous estimates of the speed of Doppler signals as the initial wave is transformed into several waves with different directions, amplitudes and frequencies. These waves are incorrectly interpreted by US equipment thus creating TA.

In the presence of large reflecting surfaces, such as calculi, the phenomenon acquires iconographic value, however adding nothing to diagnostic specificity in the presence of a rear shadow cone [2].

The usefulness of TA is enhanced in the presence of small reflective surfaces of about 2–4 mm without posterior shadow cones which make foreign bodies (FBs) difficult to detect at US grayscale imaging, whereas they can be better characterized in the presence of TA.

FBs may be introduced into the skin/muscles through lacerations and soft tissue wounds. Long-term complications of retained FBs include chronic pain and neurovascular impairment, and diagnosing and locating are therefore important in patient management and treatment.

The aim of this paper is to show how to improve diagnostic accuracy using CDUS and TA in patients experiencing discomfort due to the presence of small FBs in the soft tissues not clearly visible at US grayscale examination.

## Materials and methods

From August to October 2022 we enrolled 7 adult patients presenting with small (2–4 mm) superficial FBs located in the subcutaneous and muscle tissues, barely detectable on US grayscale. US examination was carried out on the most painful site indicated by the patient. All patients underwent US grayscale and CDUS examinations. Informed written consent, for processing personal data, was obtained from all patients. IRB approval is not required for our study.

Two radiologists, each with more than 5 years of experience in ultrasound examination, performed US Gray Scale and color Doppler ultrasound on each patient, looking for the artifacts, using a Samsung RS80A with Prestige (with a 4–15 MHz linear probe).

First, the presence of foreign bodies was investigated on the affected site using US Gray Scale, then, CD examination was performed searching the TA or the RA, that was used to verify the presence and localize the FB.

During the scanning process, the focus was placed slightly deeper than the foreign body, the gain setting was controlled, and the pulse repetition frequency (PRF) was decreased.

Patients who presented symptoms that limited daily or work activities, underwent surgery to remove the foreign body.

## Results

**Fig. 1:**
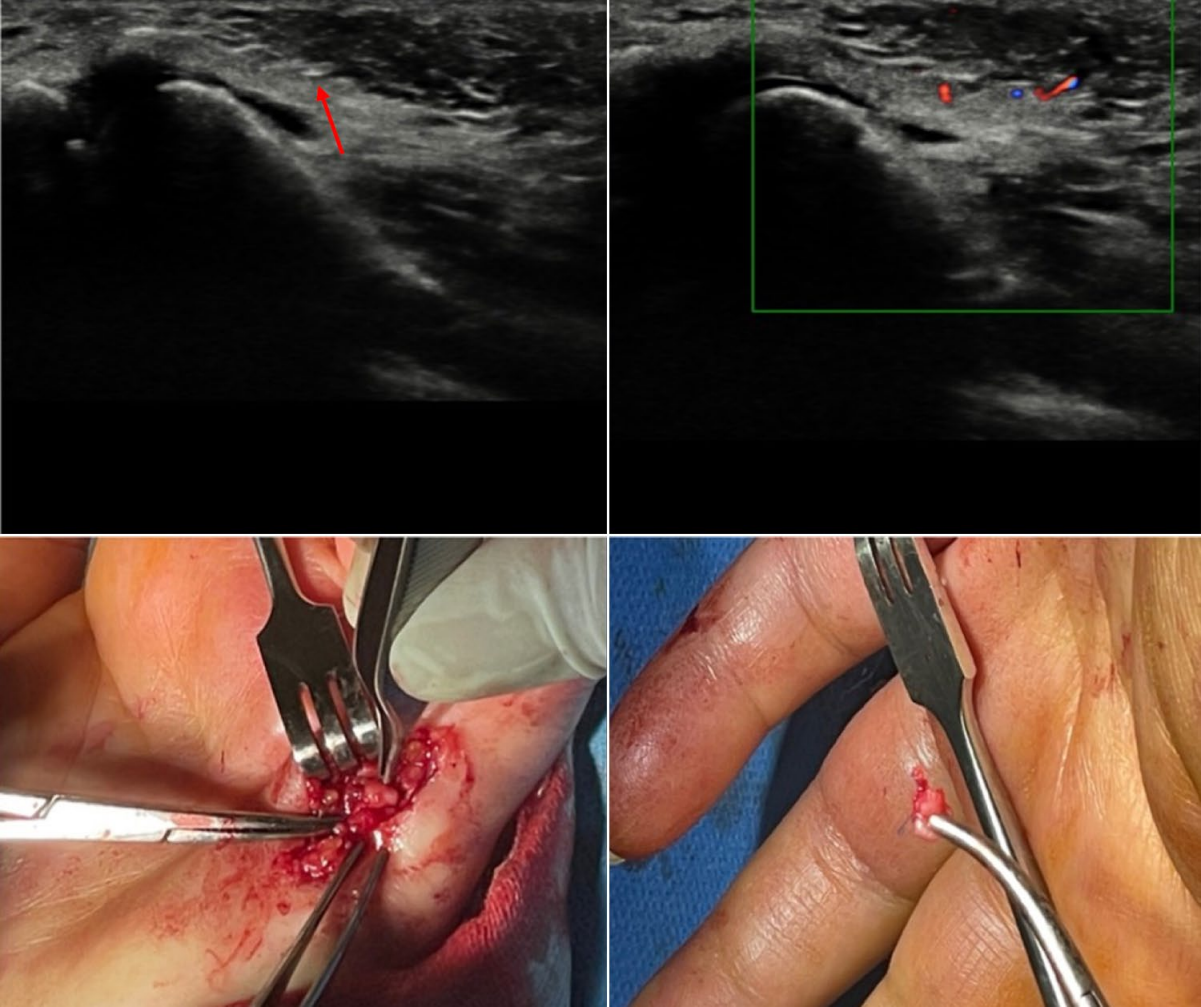
Case 1. A 39-year-old woman was admitted to our hospital because she had cut the palm of her left hand with a colored glass. She had tried to remove the small debris from the wound but later she began to experience paresthesia of the fifth finger of her left hand. US grayscale imaging was carried out where the patient felt pain and showed a small hyperechoic spot which could be observed with great difficulty at the level of the palmar surface of the hand close to the metacarpo-phalangeal joint of the fifth finger. CDUS was performed and TA indicated the presence and position of an FB. The patient experienced symptoms which limited the functionality of her hand, and she therefore underwent surgery. The surgeon removed an FB of about 3 mm near the palmar digital nerve

**Fig. 2:**
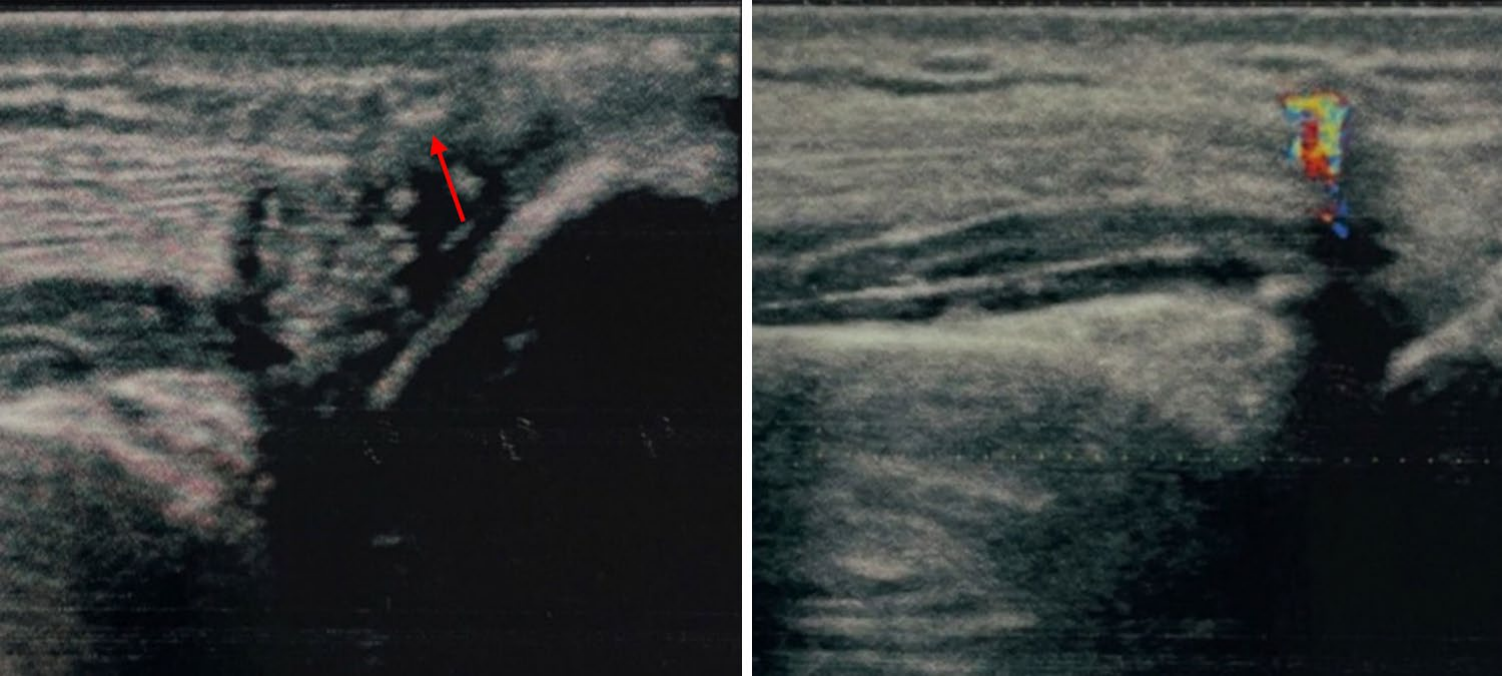
Case 2. A 25-year-old man was admitted to our hospital with a splinter of glass in the subcutaneous area of the palmar surface of the right hand. The splinter was barely visible on US grayscale as a small plaque of about 4 mm maximum size. In this case TA confirmed the presence and location of the FB

**Fig. 3:**
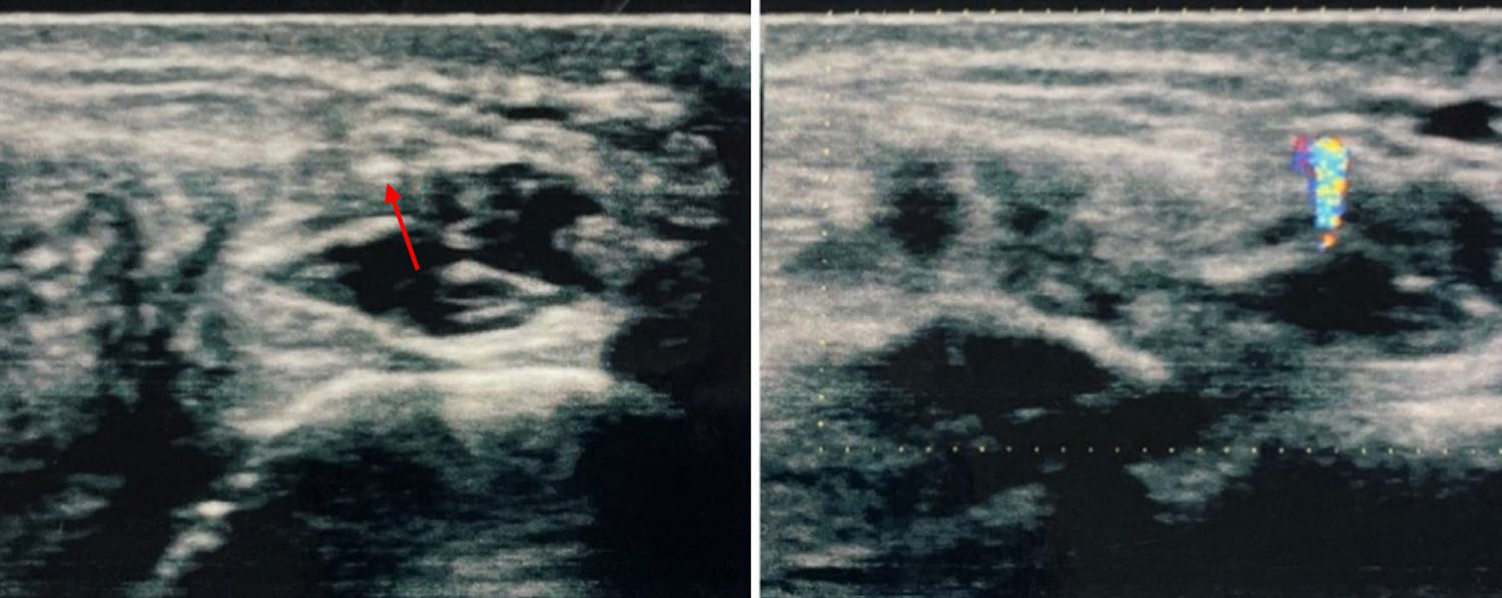
Case 3. A 36-year-old man was admitted to our hospital with a piece of metal in the muscle of the left arm. The FB was difficult to detect at US grayscale imaging but clearly visible at CDUS thanks to the presence of TA

**Fig. 4:**
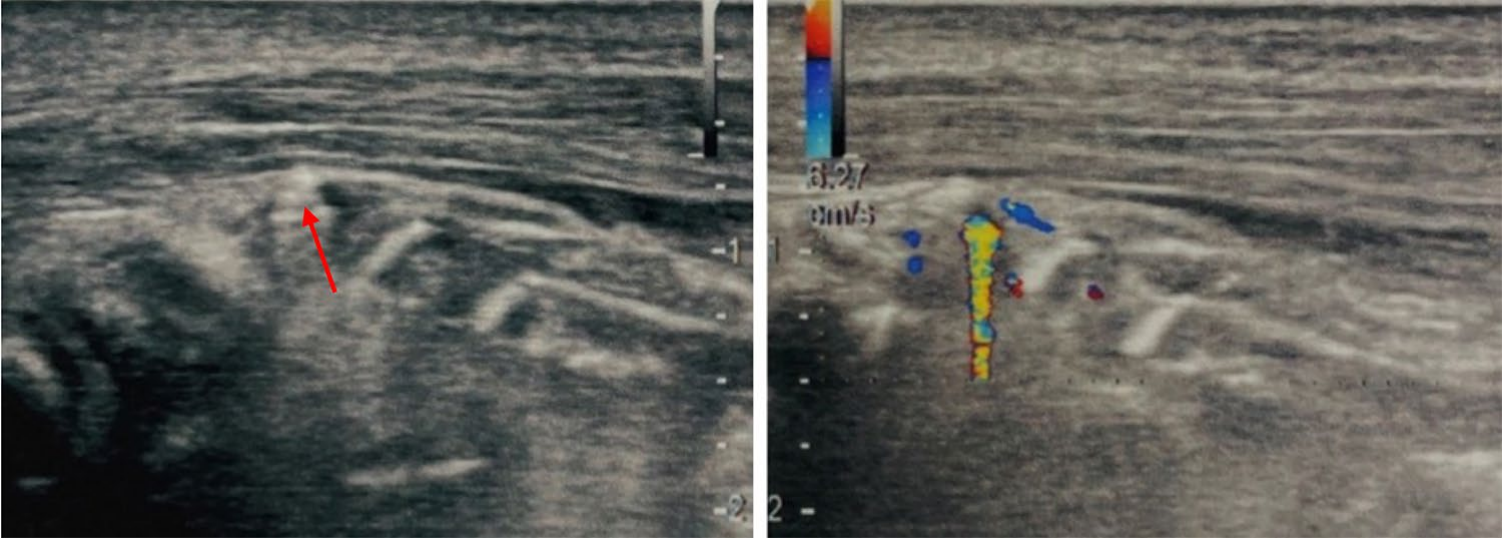
A 45-year-old woman underwent surgery and was later admitted to our hospital reporting discomfort at the level of the sutured wound. The polyethylene threads were clearly visible at US grayscale imaging whereas the small FB was not. In this case both sutures and FB produced TA. The localized FB was subsequently surgically removed, resolving the patient's symptoms

**Fig. 5:**
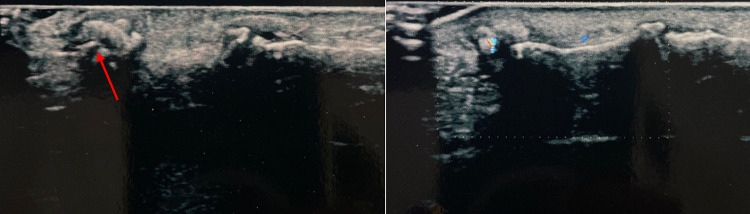
Case 5. A 47-year-old man was admitted to our hospital with pain in the palm of his right hand, which had suffered injury. The patient reported that he had cut his hand on a glass jar. Once the wound had healed, the patient began to experience pain which increased when subject to pressure. US grayscale image showed no sign of an FB, which was clearly visible at CDUS thanks to the presence of TA at the base of the fifth finger

**Fig. 6:**
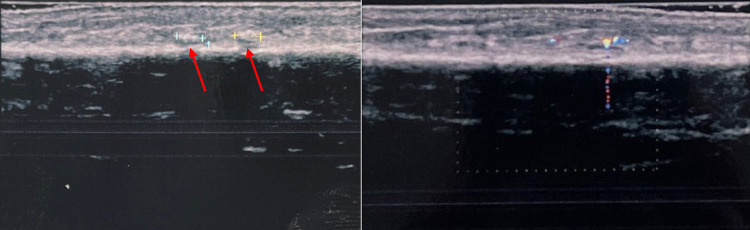
Case 6. A 27-year-old man was admitted to our hospital with discomfort in the right leg. He reported that he had hit his leg several times falling on the ground. The discomfort was very intense, and in the portion which was most painful on palpation US grayscale image showed two small hyperechoic areas that merged with the echogenicity of the surrounding subcutaneous soft tissue. In this case, CDUS showed the presence of small FBs clearly evidenced by TA

**Fig. 7:**
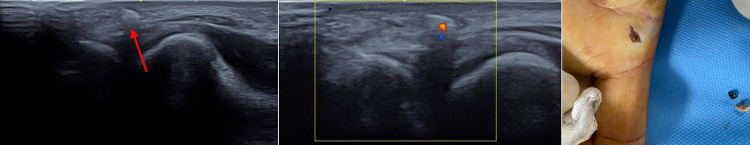
Case 7. A 55-year-old man was admitted to our hospital with pain in the palmar region of his left hand. The patient reported having had a work accident in which he injured his hand with a metal plate. Because of the material with which the patient was injured, X-ray of the hand was performed showing a radiopaque formation projecting ventrally and laterally with respect to the metacarpophalangeal joint of the second finger. For a better localization, US grayscale imaging was performed showing a tenuous hyperechoic streak below which the TA was clearly visible. Subsequently the patient underwent surgery and removal of the FB

All 7 patients examined had a foreign body of 2–4 mm, not clearly visible at the US Gray Scale, diagnosed thanks to with the presence of the artifacts. For an extended and complete description of the cases, please refer to figures below (Figs. 1, 2, 3, 4, 5, 6 and 7).

## Discussion

In a study reported by Mitchell DG, TA appeared as a rapidly changing mixture of red and blue behind presumed calcifications. The color change was not in any identifiable order, and the phenomenon was inexhaustible [2]. The spectrum was composed of close vertical bands with no outer wrapping, and the audio mode displayed treble squeaks. The color TA was found on 42 out of 140 color Doppler US images of presumed calcifications.

In vitro experiments reported by Aytac SK and Ozcan H showed that TA was generated by granular structures including sodium chloride, iron filings, emery paper, and ground chalk [3].

TA is due to a signal of slow movement of the tissue underneath the reflecting surface caused by the positioning of the probe on the skin. This signal is picked up by the probe and imperfectly eliminated by the noise elimination filter thus creating the complex beam of multiple waves that bounce between the various components of the interface and deepen in an area shielded by the reflectance of the interface itself.

In addition, erroneous estimates of the speed of Doppler signals may contribute to the genesis of TA [2, 3].

TA is an inconstant phenomenon varying in relation to two main factors, i.e. the anatomical and instrumental factors. The anatomical factor because TA is increasingly evident the more irregular the impact surface of the US beams is. The instrumental factor occurs in new devices with latest generation CDUS systems, where TA is diagnostic in over 95% of urinary stones [4].

In the presence of large reflecting surfaces, the phenomenon takes on an iconographic value due to the suggestiveness of the images but it adds nothing or little to the diagnostic specificity linked to the presence of the posterior shadow cone that is a US pathognomonic semeiological sign of highly reflective calcified structures [5].

TA is useful in the presence of small reflective surfaces (2–4 mm) without a posterior shadow cone (microcalcifications), making it possible to characterize their calcified nature, and in the presence of small FBs, which are poorly or not visible at grayscale US imaging.

TA was present regardless of the velocity (range, 1.0 cm/s–1.5 m/s), wall filter setting (100–800 Hz), probe frequency, focal depth, probe-to-interface distance (up to 21 cm) and color Doppler system [4, 6]. However, TA can be enhanced by placing the fire below the reflecting granular surface and decreasing PRF [6].

Thanks to TA, CDUS may be a reliable and non-invasive technique for evaluating and confirming the presence of small irregular or smooth reflective surfaces of 2–4 mm, not or barely visible at grayscale US, thereby providing useful information on the localization of FBs and increasing the detection of calcifications [7].

In conclusion TA is useful in the evaluation of subcutaneous and muscular FBs and provides information on their location, depth and shape, which is useful if surgical excision is required.

TA is also useful to confirm the presence of small calcifications in case of diagnostic doubt, when grayscale US does not allow a reliable visualization.

During our study, we observed that some materials generated a better visualization of TA than others. Glass proved to be a strong determinant of TA whereas wood exhibited TA to a lesser extent.
